# How to identify water from thickener aqueous solutions by touch

**DOI:** 10.1098/rsif.2011.0577

**Published:** 2011-11-09

**Authors:** Yoshimune Nonomura, Taku Miura, Takaaki Miyashita, Yuka Asao, Hirokazu Shirado, Yasutoshi Makino, Takashi Maeno

**Affiliations:** 1 Department of Biochemical Engineering, Graduate School of Science and Engineering, Yamagata University, 4-3-16 Jonan, Yonezawa, Japan; 2 Graduate School of System Design and Management, Keio University, 4-1-1 Hiyoshi, Kohoku-ku, Yokohama, Japan

**Keywords:** human skin, water, biotribology, friction, finite-element analyses, tactile display

## Abstract

Water detection is one of the most crucial psychological processes for many animals. However, nobody knows the perception mechanism of water through our tactile sense. In the present study, we found that a characteristic frictional stimulus with large acceleration is one of the cues to differentiate water from water contaminated with thickener. When subjects applied small amounts of water to a glass plate, strong stick-slip phenomena with a friction force of 0.46 ± 0.30 N and a vertical force of 0.57 ± 0.36 N were observed at the skin surface, as shown in previous studies. Surprisingly, periodic shears with acceleration seven times greater than gravitational acceleration occurred during the application process. Finite-element analyses predicted that these strong stimuli could activate tactile receptors: Meissner's corpuscle and Pacinians. When such stimuli were applied to the fingertips by an ultrasonic vibrator, a water-like tactile texture was perceived by some subjects, even though no liquid was present between the fingertip and the vibrator surface. These findings could potentially be applied in the following areas: materials science, information technology, medical treatment and entertainment.

## 1. Introduction

How do we recognize water through our five senses? For several decades, researchers have attempted to address this question [1–6] because obtaining water is one of life's most important activities. Recent neurophysiological studies have shown that humans perceive water as a sweet taste [7]. Almost all studies have focused on water perception by taste; however, humans can also recognize water by their tactile sense. Kajimoto and co-workers [8] found that mechanical stress on skin hair plays a major role in the perception of a liquid surface. We showed that water caused a stick-slip feel when a small amount was rubbed using a fingertip on artificial skin that mimicked the structure of human skin [9,10]. The results of frictional analyses predicted that this stick-slip feel was caused by a drastic change in frictional resistance. Such stick-slip phenomena and an increase of friction force have been observed on wet skin surfaces [11–26].

Living beings obtain important information from tactile stimuli through active movement. By means of active touch, much of the surrounding environment can be perceived in the absence of vision [27]. During active tactile sensation by rodents, whisker movements across surfaces generate complex whisker micro-motions that carry information about surface properties [28]. To illustrate the mechanisms of tactile detection, not only forces on the skin, but also tactile behaviour or movement velocity must be evaluated because tactile sense depends on these factors [29,30]. Furthermore, activation of sensory receptors induced by external stimuli is important because all tactile sensation originates from perceptual information conveyed by these receptors. In human skin, there are four tactile receptors: Merkel's discs, Meissner's corpuscles, Ruffini endings and Pacinians [31,32]. Each of these four receptors mediates specific portions of the overall threshold-frequency range [33].

**Figure 1.**
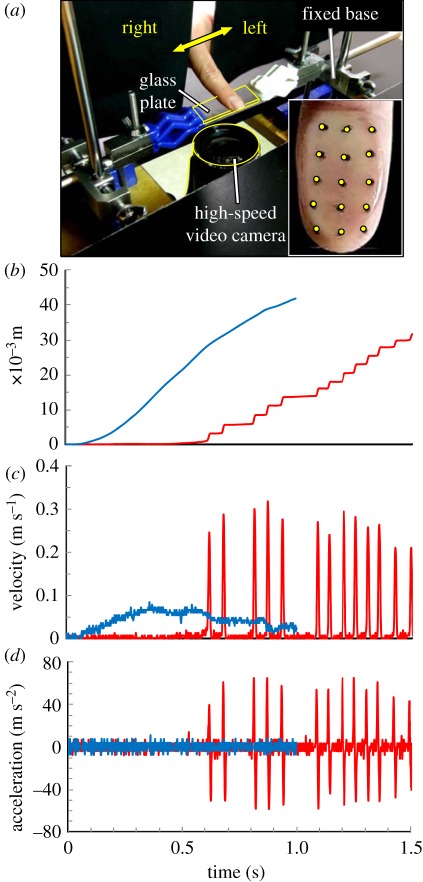
Movement behaviours of a fingertip when a subject tested water or thickener solutions. (*a*) A photograph of an observation system with a high-speed camera; examples of (*b*) movement distance, (*c*) velocity and (*d*) acceleration for water (red lines) and 2 wt% thickener solution (blue lines).

In the present study, 10 subjects identified whether the liquids on glass plates were water or thickener aqueous solutions based on their tactile sense. During the identification process, the movement velocity of the fingertips was evaluated by a high-speed camera (figure 1*a*). On the other hand, using strain gauges on two leaf springs, the friction metre measured friction and vertical forces when subjects applied liquid samples to the glass plate [34,35]. These experimental evaluations would show the mechanical stimuli on skin surface and fingertip movement when the subjects touched water. Next, we simulated the stress distribution around the tactile receptors that occurred when the subjects applied water to a glass substrate with a fingertip. The strain energy density estimated by the finite-element model correlates with the frequency of nerve impulses at tactile receptors [36–38]. We also confirmed that the strain energy density obtained by the present model is proportional to the frequency of nerve impulses in the previous study [39–41]. Furthermore, the tactile texture of water on the glass plates was displayed using a tactile display system equipped with an ultrasonic vibrator. Suitable conditions for the tactile display would reflect the characteristics of the tactile texture of water. Shiokawa *et al*. [42], Winfield *et al*. [43] and Biet *et al*. [44] showed that a vibrating plate with a squeeze film is suitable for a haptic interface. The water-like tactile texture can arise from the mechanical stimulus because the characteristic friction phenomena are the most important factor of the tactile texture of water [9,10].

## 2. Material and methods

### 2.1. Materials

Water was purified using a DX-15 demineralizer (Kurita Water Industries Ltd., Tokyo, Japan). The thickener Polyquaternium-10 (O-(2-hydroxy-3-(trimethylammonio) propyl) hydroxy cellulose chloride, POIZ C-60H) was obtained from Kao Co. (Tokyo, Japan). The aqueous samples, 0.15, 0.5, 1 and 2 wt% thickener solutions, were prepared using a Vortex Genie 2 mixer from Scientific Industries Inc. (NY, USA). These evaluation items were selected based on a preliminary test performed by three professionals: one was a researcher who had engaged in tactile evaluations for more than 10 years, and the other two were students who had studied tactile sense. The professionals stated that these thickener solutions were suitable for the items because the addition of a small amount of the thickener induced a drastic change on the tactile texture of water. Samples were heated at 60°C to dissolve the Polyquaternium-10. The thickener ingredient was checked for human safety. The viscosities of the aqueous samples are as follows: 0.9 (water), 4.5 (0.15 wt% thickener solution), 110 (0.5 wt% thickener solution), 730 (1 wt% thickener solution) and 3400 mPa s (2 wt% thickener solution). The viscosity range of these thickener solutions covers many liquids that we touch in our daily life; for example, ethyl alcohol = 1.2 mPa s at 20°C; olive oil = 84 mPa s at 20°C; machine oil = 661 mPa s at 16°C, 127 mPa s at 38°C; glycerin = 954 mPa s at 25°C [45].

### 2.2. Tactile evaluations

Tactile evaluations and friction evaluations were carried out simultaneously as follows [9,10]. Similarity with a standard sample (water), and seven tactile factors, including ‘a cool feel’, ‘a fresh feel’, ‘a slippery feel’, ‘a sticky feel’, ‘a slimy feel’, ‘a rough feel’ and ‘a stick-slip feel’ were evaluated for water and thickener aqueous solutions when subjects applied them to the glass plate installed on a friction evaluation system [9,10]. The tactile evaluations were rated on a seven-point scale, where score 7 means ‘exactly the same texture as water’, while score 1 means ‘exactly the opposite texture from water’. All evaluations were conducted according to the principles expressed in the Declaration of Helsinki. The subjects included five male students and five female students ranging in age from 20 to 23 years. The evaluations were carried out in a quiet room at 25°C ± 1°C after the subjects washed both hands with commercial liquid hand soap. The relative humidity was 50 ± 3% in the room. Subjects used their forefingers to rub 0.1 ml of a standard sample (water) followed by 0.1 ml of one of the evaluated samples on a glass plate. After filling in a questionnaire, the subjects washed their hands with water again. The unit process (i.e. applying the aqueous samples, filling in the questionnaire and washing the hands) was repeated for all samples. The order of the samples was random to eliminate order effects. During the evaluation, the composition of the samples was not revealed to the subjects. The subjects touched the liquid samples on the glass plate through a black-out curtain. The content of the test was announced previously. The subjects decided for themselves whether they would join our evaluation test.

### 2.3. Frictional evaluation

In the present study, we used a friction evaluation system that simultaneously evaluated tactile sensation and friction properties [9,10,34,35]. This device measured the friction and vertical forces using strain gauges on two leaf springs. In the present paper, the friction coefficient is defined as the ratio friction force/vertical force. The detection limits of the friction and vertical forces were 0.20 N and 0.08 N, respectively. The maximum measurable load of the device was 5 N, with a time resolution of 0.5 ms.

### 2.4. Fingertip movement analysis

We observed fingertip movements with a high-speed camera, as shown in figure 1*a*. The high-speed images were taken using an EX-F1 high-speed video camera (Casio, Tokyo, Japan) with a frame rate of 600 frames s^−1^ and a space resolution of about 200 µm pixel^−1^. Fifteen black dots of 1 mm in diameter were plotted in oil-based ink at intervals of approximately 4 mm to follow the movement. The skin surfaces on the glass plates were illuminated with a video light VL-G151 (lamp: halogen 150 W; colour temperature: 3075 K, LPL Co., Tokyo, Japan). Movement distance, velocity and acceleration of the fingertips were analysed using the two-dimensional movement analysis software Move-tr/2D v. 7.0 (Library Co., Tokyo, Japan) by the centre of gravity method. In the analysis, the values of movement for the centred dot were selected as representative values because significant differences were not observed between the movements of the 15 dots. The measurement error of the movement analysis reflects on the noises of velocity and acceleration. The noises of the velocity and acceleration were about 0.025 ms^−1^ and 20 ms^−2^, respectively.

### 2.5. Simulations using the finite-element method

The stress distribution around tactile receptors in the skin was simulated using a previously reported method [39,41]. We analysed finger deformation using the finite-element analysis software MARC (MSC Software Co., CA, USA). Electronic supplementary material, figure S1 shows a mesh model of a finger section that mimics the structure of an index finger. The plane strain element is used because the deformation outside the modelled plane is negligible nodes at the surfaces of the nail and bone. The finger skin consists of stratum corneum, epidermis, dermis and subcutaneous tissue. In the model, the stratum corneum is divided into two layers, i.e. a soft inner layer and a hard outer layer to reflect the heterogeneity of the stratum corneum because the inner layer is more hydrated than the outer layer [46]. Some measurements predicted that the hydration softens the stratum corneum layers [47]. There are papillae at the interface of the epidermis and dermis underneath the epidermal ridges. The nail and bone were not modelled because their Young's moduli are large compared with that of the skin. The four symbols in the figure represent the nodes where the four tactile receptors are located. The physical factors of these biological tissues are shown in electronic supplementary material, table S1; for example, longitudinal elastic moduli of the outer stratum corneum, inner stratum corneum, epidermis, dermis and subcutaneous tissue were 0.816, 0.408, 0.136, 0.080 and 0.034 MPa, respectively. These factors were determined based on the measured values of skins for human and guinea [39–41]. In the present simulations, the process consisted of two steps; in the first step, the finger model was moved 0.5 mm vertically towards the solid substrate for 0.125 s, whereas in the second step, it was moved horizontally for 0.875 s.

In the present simulations, the input factors that change for water and four thickener solutions were the friction coefficient and the movement velocity, which were determined from experimental results. On the basis of the experimental data, the friction coefficients were 0.84, 0.54, 0.29, 0.20 and 0.13 for water, and 0.15, 0.5, 1and 2 wt% for thickener solutions, respectively. The movement velocities of horizontal and vertical motions are shown in electronic supplementary material, figure S2. The velocity profiles were sinusoidal in acceleration/deceleration processes. Strain energy densities were filtered in consideration of the frequency characteristics of the tactile receptors. Partial fraction decomposition and inverse Laplace transform were carried out for the transfer functions of each receptor H(*s*) to obtain the time-varying function h(*t*). The filtered strain energy densities were obtained by the convolution. The filtering properties used for each tactile receptors were obtained from a previous study [41].

### 2.6. Tactile display of water texture

Mechanical stimuli were applied to human skin by a tactile display system equipped with a Langevin-type ultrasonic vibrator (SEDECO Co., Tokyo, Japan), an analogue I/O terminal (AIO-160802AY-USB, CONTEC Co., Osaka, Japan), a differential function generator (Wave Factory WF1946A, NF Co., Yokohama, Japan) and power amplifer (HSA4011, NF Co., Yokohama, Japan; electronic supplementary material, figure S3) [42]. The frequency and maximum amplitude were 28.20 kHz and 20 µm, respectively. In our previous studies, the mechanical stimuli under these conditions were suitable to raise some tactile feels: bumpy/flat, rough/fine and hard/soft feels [42]. The width of the contact surface was 30 mm. As shown in electronic supplementary material, figure S3, the mechanical stimuli were applied on skin surface intermittently to reflect stick-slip phenomena on the glass. The amplitude was controlled with a pick-up coil sensor. The position of a finger was evaluated by an infrared (IR) marker, and an IR sensor was used to control the ultrasonic vibrations. The ultrasonic vibrator was switched on and off in conjunction with the moving distance determined with an IR marker [42]. We studied the effects of the term T, which was a total movement distance in an on-period and an off-period, and the duty ratio *τ* on the tactile texture when (i) duty ratio *τ* = 0; (ii) *T* = 1 mm; *τ* = 0.5; (iii) *T* = 10 mm, *τ* = 0.5; (iv) *τ* = 1. If the velocity of the finger is assumed to be about 0.1 ms^−1^ based on the results of the fingertip movement analysis (electronic supplementary material, table S2), the friction forces change at 10 or 100 ms intervals. This interval roughly agrees with the interval of stick-slip motion when a subject touched water. Ten subjects evaluated the similarity of the ultrasonic vibrator oscillating under various conditions with standard samples (water and 2 wt% thickener aqueous solution). The tactile evaluations were rated on a seven-point scale: a score of seven indicated ‘exactly the same texture as the standard sample’, whereas a score of 1 indicated ‘exactly the opposite texture of the standard sample’.

## 3. Results

### 3.1. Effects of friction and fingertip movement on tactile feels

Similarity with a standard sample (water), and seven tactile factors were evaluated for water and 0.15, 0.5, 1 and 2 wt% thickener solutions, when 10 subjects applied them to the glass plate. Electronic supplementary material, figure S4a shows the similarity scores of the five aqueous samples. The evaluation value of water was 6.60 ± 0.49, which was the highest among the five samples. Here, the values following ± are the standard deviations. The correlations between the similarity score and the other tactile evaluations were analysed based on the correlation coefficients, *r*. Of the seven factors, the stick-slip feel had the highest value, *r* = 0.841, while the sticky feel showed a strong negative correlation, *r* = −0.824. As shown in electronic supplementary material, figure S4b, the score of stick-slip feel (slimy feel) decreased (increased) with the thickener concentration. These results indicate that the stick-slip feel is the characteristic property of the texture of water on a glass plate [9,10].

Live images of subjects applying water or the thickener aqueous solutions were obtained with a high-speed video camera. Slow-motion images (600 frames s^−1^, 20 times slower than normal speed) are shown in the electronic supplementary material (videos S1 and S2). In the case of water, a regular pattern consisting of ‘stick periods’, when the fingertip remained stationary and ‘slip periods’, when it moved rapidly, was repeated regardless of the subject's motion. Figure 1*b* shows the examples of movement distance, velocity and acceleration of the fingertip. The profile of the movement distance (*x*) was a staircase pattern in which *x* increased discontinuously at several tens of millisecond intervals. During the slip periods, the average velocity and acceleration of the 10 subjects were 0.231 ± 0.076 m s^−1^ and 66.0 ± 16.4 m s^−2^, respectively (electronic supplementary material, table S3). Surprisingly, the acceleration was about seven times greater than gravitational acceleration. During the slip periods, frictional stimuli with a friction force of 0.46 ± 0.30 N and friction coefficient of 0.84 ± 0.29 were applied to the fingertips at several tens of millisecond intervals (electronic supplementary material, figure S5 and table S2). These stimuli caused a stick-slip feel, i.e. an intermittent friction feel, in eight of 10 subjects. In our knowledge, it is the first report on the high-speed observation when subjects identify water on solid substrates based on information from tactile stimuli through active movement.

Such stick-slip motion of the fingertips was not observed when the subjects applied aqueous solutions containing thickener (electronic supplementary material, video S2); instead, the fingertips slid on the glass plate smoothly without a change in acceleration (figure 1*b*). Electronic supplementary material, table S3 shows the effect of thickener concentration on the times and acceleration of stick-slip motions. These observed values (183 ± 58 and 66.0 ± 16.4 m s^−2^, respectively, for water) decreased with increasing thickener concentration. The friction properties of human skin have been studied *in vivo*. As mentioned in §1, the friction depends on the hydration condition of human skin [11–26]. On human skin, a glass slider was observed to exhibit a stick-slip motion that may be attributed to the accumulation of a water film, which becomes more pronounced with increasing sliding velocity [17].

### 3.2. The stress distribution around tactile receptors

**Figure 2.**
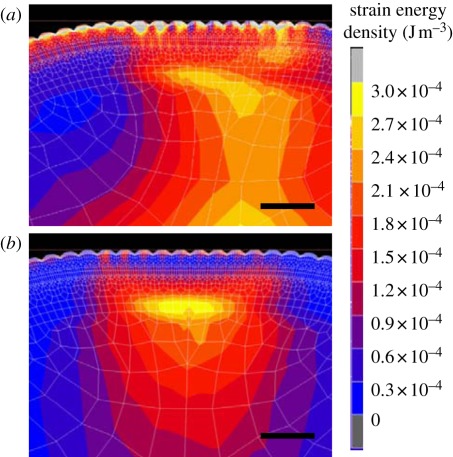
(*a*) Distribution of strain energy density under the skin surface when a subject applied water or (*b*) 2 wt% thickener solution. Scale bars, 1.5 mm.

To show the effects of friction stimuli with acceleration on neural systems, the stress distribution around the tactile receptors in the skin was simulated using the finite-element method. The strain energy density obtained by the simulation reflected the firing frequency. Amplitudes greater than a specific value produced one impulse every cycle [48]. The strain energy distribution images show a model finger press a rigid object 0.5 mm vertically for 0.125 s and then move it horizontally for 0.875 s (figure 2, electronic supplementary material, videos S3 and S4). For water, the energy density profile showed the spatio-temporal asymmetric properties with the strain energy concentrated in the direction of forward motion and periodic changes at several hundred millisecond intervals. In contrast, the energy density profile showed the symmetric properties for 2.0 wt% thickener aqueous solution.

**Figure 3.**
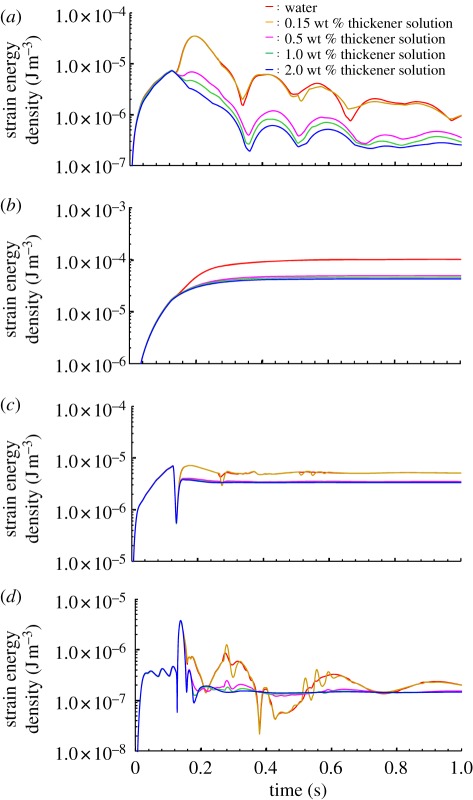
(*a*) Strain energy density at Meissner's corpuscles, (*b*) Merkel discs, (*c*) Ruffini ending and (*d*) Pacinians when a subject applied water and thickener solutions on glass.

These energy profiles were filtered based on the response characteristics of each tactile receptor [39–41]. Figure 3 shows temporal changes in strain energies on the tactile receptors when subjects touched water or thickener aqueous solutions. Contact with these liquids excited all tactile receptors, and unusual patterns were induced on Meissner's corpuscles and Pacinians following the application of water. Namely, the strain energy on Meissner's corpuscles was 4 × 10^−5^ J m^−3^ at 0.2 s and decreased with periodic changes at approximately 100 ms intervals. Additionally, the energy on Pacinians was 4 × 10^−6^ J m^−3^ at 0.15 s and changed at intervals between several tens of millisecond and 100 ms. These characteristics of the strain energy profiles were abolished by increasing the thickener concentration. Although the strain energy was distributed on Merkel's discs and Ruffini endings, an unusual profile was not observed for water. These results predict that the acceleration, which was seven times greater than gravitational acceleration, could activate Meissner's corpuscles and Pacinians in predictable patterns. In our knowledge, this is the first report on the effects of mechanical stimuli induced by the contact with wet substrates on neural systems.

### 3.3. Display of water-like tactile texture

**Figure 4.**
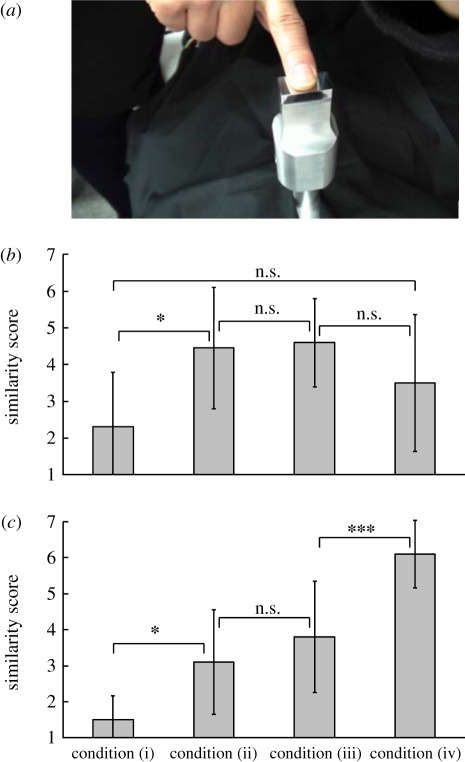
Tactile stimulation of a fingertip by an ultrasonic vibrator. (*a*) Photograph of the ultrasonic vibrator. (*b*) Similarity of the tactile texture induced by the ultrasonic vibrator to that of water or (*c*) 2 wt% thickener solution. The ultrasonic vibrator oscillated under the following conditions: (i) no oscillation; (ii) wavelength *T* = 1 mm, duty ratio *τ* = 0.5; (iii) *T* = 10 mm, *τ* = 0.5; and (iv) *τ* = 1. Error bars denote standard deviations. The statistical significance was measured between similarity scores under four conditions (i)–(iv). The symbols single asterisk (*), triple asterisks (***) and n.s. mean *p* < 0.01, *p* < 0.001 and non-significant in *t*-tests, respectively.

Mechanical stimuli were then applied to human skin using a tactile display equipped with an ultrasonic vibrator to mimic the texture of water. In this system, the friction force between the fingertip and the contact surface changed with the oscillation amplitude of the ultrasonic vibrator [42–44]; the friction force on the oscillating vibrator was lower than that on the resting vibrator. The squeeze effect of ultrasonic vibration decreases the friction force. The similarity score with water was 2.3 ± 1.5 when the vibrator did not oscillate, and 4.5 and 4.6 when the vibrator oscillated at a term *T* of 1 mm or 10 mm, respectively (electronic supplementary material, figure S4). The highest similarity score was obtained when the vibrator oscillated intermittently. In addition, the similarity score for the 2 wt% thickener solution was highest when the duty ratio *τ* was 1, i.e. when the vibrator was oscillating continuously. These results demonstrated that a water-like tactile texture could be achieved by applying shear force with a strong acceleration per several tens of millisecond. The similarity score of 4.6, however, was not statistically significant. This dissociation may arise from the fact that thermal sensation and tenderization of the skin and irregularity of the stick-slip pattern were neglected in the present system [49,50] (figure 4).

## 4. Discussion

On the basis of the earlier mentioned results, we propose that the detection mechanisms that humans distinguish water from thickener aqueous solutions by the stick-slip motions with large acceleration per several tens millisecond and the firing of Meisner's corpuscles and Pacinians. The present mechanism is supported comprehensively by the results of three experiments and simulations: the frictional evaluation, movement analysis, finite-element analysis and tactile display. For example, the stick-slip motion with large acceleration was observed by both the frictional evaluations and fingertip movement analysis. The results of tactile display backed up the importance of the stick-slip motion to detect water. The experimental results of the frictional evaluation and movement analysis also contributed to show realistic stress distribution on tactile receptors in the finite-element analysis.

The application of a shear force with acceleration seven times greater than gravitational acceleration was characteristic for water and was abolished by the addition of a small amount of thickener. The quantitative analysis of finger movement on wet glass plate is achieved by our high-speed observation. This intermittent stimulation is caused by the stick-slip phenomenon, which is well known in the field of tribology. It is characterized by the phenomenon transitions between a static (solid-like) state and a kinetic (liquid-like) state and is observed when lower sliding velocity induces a larger frictional resistance [51]. Water swells human skin and increases the contact area and friction force between skin and solid surfaces [17,20]. Recently, André *et al*. [25] reported that the skin hydration level markedly affected the dynamics of the contact encapsulated in the course of evolution from sticking to slipping. In contrast, stick-slip motion is inhibited by thickener in contaminated water [12]. Adams *et al*. showed that this intermittent motion may be attributed to the accumulation of a water film, which becomes more pronounced with increasing sliding velocity [17,26]. Such accelerations are common during tactile interactions; Maeno *et al*. [52] proposed a method for controlling a grasping force using the strain distribution in relation to the stick/slip information at the surface of the elastic finger. Shao *et al*. [53] predicted stress oscillations during sliding over a textured surface.

Is our model that water is perceived by the firing of Meissner's corpuscles and Pacinians reasonable? Meissner's corpuscles are responsible for the perception of events that produce low-frequency, low-amplitude skin motion and detect microscopic skin motions. Pacinians are responsible for the perception of events transmitted to the hand as high-frequency vibrations and are sensitive to acceleration [54]. It is reasonable that Pacinians respond to the stick-slip motion per several tens of millisecond because they are sensitive to vibrations of several hundred hertzs. The argument that Meissner's corpuscles receptors are slip detectors is based on the experiments by Srinivasan *et al*. [55], who showed that small surface features are required for slip detection and that the responses of Meissner's corpuscles account for the limits of slip detection. In this vein, the idea that water is perceived by the firing of Meissner's corpuscles and Pacinians is justified.

In the present study, we show that humans have a sophisticated system to recognize water from thickener aqueous solutions by touch: humans distinguish water from thickener aqueous solutions by the stick-slip motions with large acceleration and the firing of Meisner's corpuscles and Pacinians. These findings could be generalized when humans recognize water from other liquids that have a similar viscosity and water containing specific solutes. In previous papers, we showed that subjects can differentiate water from silicone oil, surfactant aqueous solutions and ethanol aqueous solutions whose viscosities were almost similar to water [9,10]. This finding adds to our understanding of the perception mechanisms for water, which is the main component of the human body and an essential material for life. In day-to-day life, we frequently perceive water through our tactile senses. Of course, in our real-life situations, the stick-slip motion is one of the cues to identify water from other liquid materials. For example, although tactile discrimination of water from aqueous salt/alcohol solutions is difficult [9], we can distinguish them with olfactory or visual cues. These findings will be useful when designing virtual reality systems to mimic the sensation of the texture of liquids.
